# The Role of Hysteroscopy for the Treatment of Interstitial Ectopic Pregnancy: A Systematic Review

**DOI:** 10.3390/jcm15062158

**Published:** 2026-03-12

**Authors:** Guglielmo Stabile, Laura Vona, Stefania Carlucci, Francesco Nappi, Stefania Biffi, Anna Pitsillidi, Stefano Restaino, Giuseppe Vizzielli, Luigi Nappi

**Affiliations:** 1 Department of Medical and Surgical Sciences, Institute of Obstetrics and Gynaecology, University of Foggia, 71122 Foggia, Italy; guglielmost@gmail.com (G.S.); s.carlucci86@gmail.com (S.C.); luigi.nappi@unifg.it (L.N.); 2 Department of Medicine, “G. D’annunzio” University of Chieti-Pescara, 66100 Chieti, Italy; francescoluigi.nappi@gmail.com; 3 Institute for Maternal and Child Health IRCCS “Burlo Garofolo”, 34100 Trieste, Italy; stefania.biffi@burlo.trieste.it; 4 Department of Obstetrics and Gynaecology, Rheinland Klinikum Neuss, Preußenstrasse 84, 41464 Neuss, Germany; anna.pitsillidi@gmail.com; 5 Department of Maternal and Child Health, Obstetrics and Gynecology Clinic, University-Hospital of Udine, 33100 Udine, Italy; stefano.restaino@asufc.sanita.fvg.it (S.R.); giuseppevizzielli@yahoo.it (G.V.)

**Keywords:** ectopic pregnancy, interstitial pregnancy, treatment, hysteroscopy

## Abstract

**Background/Objectives:** Interstitial ectopic pregnancy is a rare and potentially life-threatening condition, accounting for 1–6% of ectopic pregnancies. Its location complicates diagnosis and management, and no standardized treatment guidelines exist. Fertility-preserving, minimally invasive approaches have been proposed as alternatives to medical therapy or radical surgery. This systematic review evaluates the safety and effectiveness of hysteroscopic treatment, focusing on uterine preservation and reproductive outcomes. **Methods:** This systematic review was conducted according to PRISMA guidelines and registered in PROSPERO (CRD420251249508). Web of Science, Scopus, and PubMed were searched from inception to January 2026. Eligible articles included case reports and case series describing interstitial pregnancies managed hysteroscopically, alone or combined with minimally invasive treatments, without medical therapy. Study quality was assessed using the JBI Checklist. **Results:** Eight studies comprising 21 patients were included. Mean gestational age at diagnosis was 55 days, and mean β-hCG level was 7981 IU/L (range 1440–32,000 IU/L). Hysteroscopic management was successful in 16 of 21 cases (76%). Five patients required rescue therapy. Reduced residual myometrial thickness was the main factor associated with treatment failure. Mean time to β-hCG normalization was 32 days. **Conclusions:** Hysteroscopic management is a safe and effective minimally invasive option for clinically stable patients with interstitial ectopic pregnancy. It allows direct visualization, targeted tissue removal, and preservation of uterine integrity and fertility, with limited morbidity. Ultrasound guidance is generally sufficient, reserving laparoscopy for high-risk cases. These findings support hysteroscopy as a fertility-preserving strategy, though larger prospective studies are needed to confirm long-term reproductive outcomes.

## 1. Introduction

The 2020 ESHRE classification standardises the terminology used to describe ectopic pregnancies on ultrasound. In this classification, interstitial pregnancy is considered a subtype of tubal ectopic pregnancy when the gestational sac implants in the portion of the fallopian tube that penetrates the uterine muscle [1]. The incidence of this condition is estimated at 1–6% of all ectopic pregnancies, with a maternal mortality rate of 2–2.5% [2]. These data identify a relatively rare ectopic pregnancy that is difficult to differentiate from a lateralised eutopic pregnancy [1]. Interstitial ectopic pregnancy has few early clinical symptoms (abdominal pain in the first trimester of pregnancy) and vaginal bleeding [3]. A diagnostic process based on clinical findings, gestational age at diagnosis, human chorionic gonadotropin (β-HCG) dosage, ultrasound features, and patient preferences are useful factors in the decision-making process regarding the best treatment [4]. Treatment may be medical, surgical, or close observation. There are no guidelines in the literature for the management of this condition. Therefore, authors prefer surgical or medical intervention based on their skills and experience [5]. This review aims to understand the outcomes of hysteroscopic treatment as a monotherapy, in combination with another minimally invasive surgical technique (e.g., suction and curettage) or under laparoscopic guide without any medical treatment. Furthermore, considering that standard management traditionally includes cornual resection or systemic methotrexate administration, this review seeks to contextualize hysteroscopy as a potentially less invasive alternative, assessing its safety and efficacy in the management of a rare and difficult-to-treat condition.

## 2. Materials and Methods

### 2.1. Search Strategy

This systematic review was conducted in accordance with the PRISMA guidelines for systematic reviews (see Supplementary Table S1 PRISMA checklist) [6]. Two independent reviewers (L.V. and G.S.) performed a comprehensive literature search of the Web of Science, Scopus, PubMed and Cochrane Central Register of Controlled Trials (CENTRAL) databases, from database inception through 31 January 2026. No publication date restrictions were applied. The search strategy combined the following keywords and MeSH terms: ectopic pregnancy, interstitial pregnancy, treatment, hysteroscopy.

**Figure 1:**
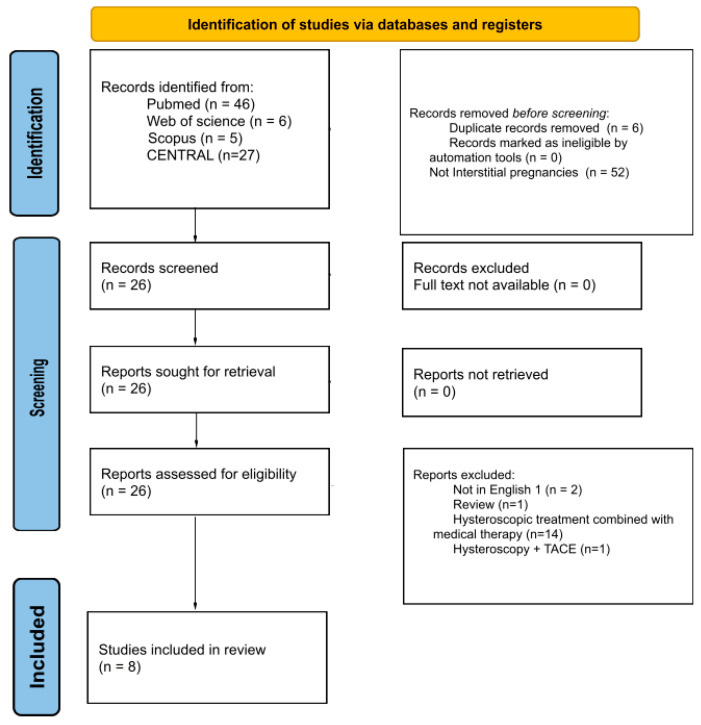
PRISMA flow diagram.

Studies in which interstitial pregnancies were treated with hysteroscopic management alone or in combination with another minimally invasive surgical intervention (suction and curettage, laparoscopic guide or laparoscopic milking) were considered. The study selection process is detailed in the PRISMA flow diagram (Figure 1).

### 2.2. Eligibility Criteria

Eligible study designs included case reports, randomized controlled trials, prospective controlled studies, prospective cohort studies, retrospective studies, and case series. Only full-text articles published in English were included. Only articles reporting cases of interstitial pregnancies treated with hysteroscopic management alone or assisted by minimally invasive surgical treatments like suction and curettage, laparoscopic guide or laparoscopic milking were included. Studies were included if they reported cases of interstitial pregnancy diagnosed according to established ultrasound criteria. Interstitial pregnancy was defined by the presence of: (1) an empty uterine cavity; (2) a gestational sac located laterally in the interstitial (intramural) portion of the fallopian tube; and (3) the presence of the interstitial line sign. When the diagnostic criteria were not explicitly detailed, studies were considered eligible if the described ultrasound findings were consistent with these features.

### 2.3. Exclusion Criteria

Systematic reviews, meta-analyses, letters to the editor, and conference abstracts were excluded. However, reference lists of relevant reviews were manually screened to identify additional eligible studies. Articles not written in English, or those including non-interstitial ectopic pregnancies, as well as cases in which interstitial pregnancies were treated with non-hysteroscopic surgical management, medical therapy or a combination of these, were excluded. The use of alternative treatments, such as interventional radiology procedures (e.g., TACE—Transcatheter Arterial Chemoembolization) [7] were also exclusion criteria.

### 2.4. Data Extraction and Risk of Bias Assessment

Two reviewers (G.S. and L.V.) independently screened all records retrieved through the database search, beginning with publication year, citation details, title, authorship, and abstract, followed by full-text evaluation. Duplicate entries were manually identified and removed prior to screening. After eliminating irrelevant studies based on titles and abstracts, the reviewers independently assessed the full texts of the remaining articles for eligibility. Any disagreements were resolved through discussion and consensus.

The methodological quality of the included studies was evaluated using the Joanna Briggs Institute (JBI) Critical Appraisal Checklist (Supplementary Table S2). The study protocol was registered in the PROSPERO database (registration number: CRD420251249508). As this review includes exclusively case reports and case series, a potential risk of bias is acknowledged.

### 2.5. Data Synthesis

Data extracted included patient demographics (age, obstetric history), method of conception, gestational age at diagnosis, serum β-hCG levels, and ultrasonographic parameters such as residual myometrial thickness, gestational sac diameter, and the presence of embryonic cardiac activity. Treatment-related variables included the type of hysteroscopic approach, the use of laparoscopic or ultrasonographic guidance, catheter size for suction, and the use of hemostatic agents. The extracted outcomes comprised treatment success, the need for rescue interventions, and time to β-hCG normalization (Table 1). Treatment success was defined as complete resolution of the interstitial pregnancy without the need for additional surgical or medical interventions, confirmed by ultrasound evidence of absence of residual trophoblastic tissue and/or a progressive decline of serum β-hCG levels until normalization.

Continuous variables were summarized as means where possible, while categorical variables were presented as frequencies or percentages. The results of individual studies were visually displayed using tables. Baseline characteristics were stratified by treatment outcome and summarized separately for treatment failure and treatment success. Given the small number of patients and heterogeneity among the included studies, some data were synthesized descriptively.

## 3. Results

### 3.1. Study Selection and Methodological Quality Assessment

The literature search identified a total of 84 records from electronic databases, including PubMed (*n* = 46), Web of Science (*n* = 6), Scopus (*n* = 5) and CENTRAL (*n* = 27). Before screening, 58 records were removed: 6 duplicates and 52 records that were not related to interstitial pregnancies. The remaining 26 records were screened, and all were sought for retrieval. No reports were excluded due to unavailability of full text. All 26 reports were assessed for eligibility. Twenty reports were excluded for the following reasons: not in English (*n* = 2), review articles (*n* = 1), hysteroscopic treatment combined with medical therapy (*n* = 14), and hysteroscopy combined with Transcatheter Arterial Chemoembolization (*n* = 1). Ultimately, eight studies were included in the final review, comprising 21 patients.

The methodological quality of the included case reports was assessed using the JBI Critical Appraisal Checklist. Overall, the studies demonstrated good methodological quality. Six of the eight reports met all eight appraisal criteria. Two studies showed unclear reporting regarding patient demographic characteristics and the description of diagnostic tests or assessment methods [8,10]. Clear reporting of the intervention, post-intervention outcomes, and adverse or unanticipated events was observed across all included studies.

### 3.2. Patients’ Characteristics and Pregnancy Data

The mean age was 31 years (range 20–42). Obstetric history was reported in six studies. The method of conception was described for 2 patients, who conceived through IVF [13,14].

The mean gestational age was 55 days (range 35–85). The mean β-hCG level was 7981 IU/L (range 1440–32,000), although two studies did not report this value [10,11].

### 3.3. Ultrasound Findings

Regarding the ultrasonographic data, the residual myometrial thickness between the ectopic pregnancy and the uterine serosa was reported in only three studies, ranging from 1 to 4 mm (mean 1.8 mm) [12,14,15]. The gestational sac diameter ranged from 5 to 67 mm (mean 40). The presence of an embryonic pole with cardiac activity was documented two studies [11,15].

### 3.4. Treatments

In four studies, laparoscopy was used as guidance to visualize the suction and hysteroscopic procedures (successful in 12 of 17 patients—71%) [8,9,10,12]. In the studies by Nezhat et al., laparoscopic milking —a technique in which the ectopic gestational tissue is gently mobilized from the fallopian tube toward the uterine cavity—was used to displace the gestational sac toward the uterine cavity prior to hysteroscopic removal, with or without suction and curettage [11,13]. In four studies, the size of the Karman Cannula used for suction was reported [8,9,10,12], and in two of these, a pediatric 8-Fr cannula was employed [8,9]. Among patients treated with the 8-Fr cannula, 6 of 9 achieved procedural success, while all 6 patients treated with 15- or 18-Fr cannulas had successful outcomes.

In the 2025 case by Liu hysteroscopic treatment was performed under ultrasound guidance alone (success rate 100%) [14]. In the case reported by Cronin et al., the gestational sac was removed hysteroscopically, followed by US guided curettage to remove the remaining tissue [15]. A hemostatic agent (Oxytocin or Vasopressin) was used by Cai et al. and Nezhat et al. [9,11].

### 3.5. Outcomes

The treatment approach was successful in 16 of 21 patients (76%). Treatment failure occurred in 5 patients (26%), all of whom required rescue management.

In four of these cases, surgical intervention was necessary: laparoscopic cornual resection was performed in three patients (two reported by Niu et al. and one by Cai et al.) [9,12], while one patient in the study by Cai et al. required laparotomic cornual resection associated with salpingectomy [9]. In the remaining case, reported by Cai et al., adjuvant methotrexate (MTX) therapy was administered because of persistent residual amnio chorionic tissue [9].

The mean time to β-hCG normalization was 32 days (range 2–63).

Table 2 and Table 3 show the baseline characteristics of patients according to treatment outcome. Table 2 reports data for patients with treatment failure, while Table 3 includes patients with treatment success, including mean age, gestational age, serum β-hCG, residual myometrial thickness (RMT), and gestational sac (GS) diameter.

## 4. Discussion

Interstitial pregnancies are a rare type of ectopic pregnancy associated with an increased risk of severe hemorrhage and maternal morbidity [2]. The optimal management for this type of ectopic pregnancy is unclear and there are not guidelines in the literature to refer to for treatment [16]. The aim of the therapy should be to terminate the pregnancy while minimizing the risk of bleeding and preserving fertility [17]. Alternative management strategies have shown variable success rates. Combination therapy with systemic methotrexate and mifepristone was effective in 63.6% of cases [18], while in a cohort of 98 patients, surgical treatment (cornual resection) was successful in 100%, intralesional single-dose methotrexate in 70.6%, and intramuscular multidose methotrexate in 31% of cases [19]. Furthermore, in cases of advanced maternal age with recourse to medically assisted procreation, the resolution time is also an important factor to consider in choosing the best management [20]. In these cases, in fact, it would be necessary to consider the need to wait 3–6 months before a new pregnancy in the case of using methotrexate for medical therapy [21]. In addition, in many cases managed with medical therapy a longest time for *β*-hCG relativization is needed [20]. According to some authors the observation of pregnancy vascularization, even if subjective, could be the leading point to choose about the possibility of a medical approach [17]. A higher vascularization could suggest the presence of a wider syncytiotrophoblast and a consequent higher progesterone secretion that leads to a minor efficacy of the therapy [22]. Syncytiotrophoblast plays the most important role in maintaining pregnancy by directly contacting the endometrium for oxygen exchange [23]. It also secretes human placental lactogen, which regulates maternal metabolism to ensure an adequate supply of nutrients to the fetus and secretes *β*-hCG to maintain the corpus luteum of the ovary [24,25]. As our results demonstrate, hysteroscopy, even when not guided by laparoscopy, is an effective tool for the resolution of this type of pregnancy. Furthermore, compared to dilatation and curettage allows direct visualization of the uterine cavity and of the pregnancy ensuring a good hemostasis coagulating and separating the chorionic villi and placental tissues from the myometrium [20]. While many included reports still describe the use of Karman cannulas and curettage, insights from more frequent cases of cesarean scar pregnancy suggest that hysteroscopic techniques provide improved visualization, targeted tissue removal, and better control of bleeding [26]. This comparison indicates that hysteroscopy may reduce the risk of incomplete removal and uterine trauma compared to purely suction methods [20]. Where feasible, the inclusion of intraoperative images can further illustrate procedural steps and support reproducibility [27].

Minimally invasive approaches, such as hysteroscopy, provide distinct advantages over more extensive surgical procedures, including cornual resection or cornuotomy, particularly with respect to perioperative morbidity and the preservation of uterine integrity and subsequent fertility. In the study by Lee et al., patients managed with cornual resection or cornuotomy, had an overall incidence of persistent interstitial pregnancy of 6.7% [28], underscoring a clinically relevant complication that appears to be largely avoidable with minimally invasive uterine-sparing techniques, as demonstrated in our cohort. Furthermore, more radical procedures are characterized by prolonged operative times and a higher risk of significant intraoperative blood loss, while laparoscopic wedge resection has been associated with serious obstetric sequelae in subsequent pregnancies, including uterine rupture or dehiscence in up to 30% of cases [29]. In contrast, hysteroscopy minimizes disruption of the myometrium, reduces surgical trauma, and preserves the structural integrity of the uterus, as the procedure is performed under direct visualization of the uterine cavity, allowing the pregnancy to be removed under hysteroscopic guidance, further contributing to the preservation of uterine architecture and potentially reducing the risk of adverse reproductive outcomes while optimizing the prospects for future gestations [30].

Patient age, history of previous abortions, and parity do not appear to affect the outcome of hysteroscopic treatment. Interestingly, gestational age at the time of intervention also does not seem to influence the procedural outcome, contrary to what might be expected. In fact, the patients in whom the treatment was effective had a longer gestational age and a larger average size of the gestational sac compared to those in whom the hysteroscopic therapy was unsuccessful. This could be explained by a simpler hysteroscopic surgical approach in patients with a more advanced gestational age, characterized by a larger gestational sac that protrudes further into the uterine cavity [31]. In contrast, at earlier stages of pregnancy, smaller gestational sacs may make the procedure more challenging due to the difficulty of the instruments in reaching the gestational sac and the trophoblast, thereby increasing the risk of bleeding and uterine perforation. Residual myometrial thickness (RMT) is typically measured by ultrasound and represents the amount of muscular tissue between the gestational sac and the serosal surface of the uterus. RMT is an important anatomical parameter for procedural planning. What our data suggest significantly influences the outcome is the RMT, and this is easy to understand if one considers that an hysteroscopy electrode or loop approximately 0.3 mm in diameter is used to remove the pregnancy [32,33]. For this reason, in the case of a reduced RMT (even just 1 mm less) the risk of uterine perforation increases significantly.

The procedural success rate does not appear to increase when laparoscopy is used as guidance compared with ultrasound during hysteroscopy. Therefore, laparoscopy should be regarded as a secondary approach, for instance in the event of active bleeding or suspected uterine perforation, rather than being routinely employed as guidance for hysteroscopy, given that ultrasound has proven to be a reliable and effective tool. Indeed, avoiding the use of laparoscopy as a guidance modality also reduces patient exposure to more invasive and potentially unnecessary procedures [34]. However, this finding may be influenced by the fact that cases managed under laparoscopic guidance were characterized by a lower residual myometrial thickness, and ultrasound guidance has been reported in only a few cases, so evidence is limited and further studies are needed.

One of the main challenges during hysteroscopy is the operator’s limited ability to accurately assess uterine wall thickness and the extent of uterine distension during the procedure [35]. In cases where the residual myometrial thickness (RMT) is less than 2 mm, laparoscopy may serve as an additional safety adjunct to enhance procedural control.

While laparoscopy does not appear to be necessary as a diagnostic modality—given the 100% diagnostic accuracy of ultrasound in the analysed cases—it may still have a role in selected clinical scenarios, particularly when the RMT is <2 mm.

Finally, the use of uterine aspiration as an adjunct to the hysteroscopic procedure appears to be a valuable adjuvant, allowing for a more rapid and effective resolution of the pregnancy. Our analysis suggests that the size of the Karman cannula may influence procedural success. In our review, patients treated with a pediatric 8-Fr cannula had a slightly lower success rate (6 of 9) compared to those treated with 15- or 18-Fr cannulas (6 of 6). Although the sample size is small, this finding may indicate that smaller cannulas could be associated with a slightly lower likelihood of complete removal of gestational tissue.

Based on these findings, we feel that the use of ultrasound can be suggested, as it is less invasive and more effective in preserving the patient’s fertility.

### 4.1. Predictors of Procedural Failure

In our review, 5 patients experienced procedural failure (26%), with four requiring laparoscopic cornual resection and one requiring methotrexate. We explored whether specific preoperative characteristics could predict failure. Although patient age, parity, and history of prior abortions did not appear to influence outcomes, cases with a reduced residual myometrial thickness (<2 mm) were more likely to experience incomplete removal. Additionally, smaller gestational sacs at earlier gestational ages may contribute to technical challenges, potentially increasing the risk of failure. While these observations are limited by the small sample size, they suggest that careful assessment of RMT and gestational sac size may help identify patients at higher risk for unsuccessful hysteroscopic management.

### 4.2. Limitations

This review is limited by the small sample size, comprising only 21 patients across eight studies, which restricts generalizability. Comparisons between different treatment techniques are particularly challenging due to the limited number of studies employing each approach. Follow-up data and long-term reproductive outcomes were limited, preventing definitive conclusions regarding fertility preservation. Future studies should aim to report procedure-specific complication rates and, where feasible, assess post-treatment tubal patency of the affected tube, as these data would provide further insights into the safety and reproductive outcomes of minimally invasive management of interstitial pregnancy. Finally, the inclusion of primarily case reports and small case series introduces the potential for publication bias and may overestimate procedural success.

## 5. Conclusions

Interstitial ectopic pregnancy, increasingly observed due to assisted reproductive technologies and advanced maternal age, remains a complex clinical entity because of its rarity, absence of standardized guidelines, and heterogeneous outcomes. Our review indicates that, in hemodynamically stable patients, hysteroscopic management represents a safe, effective, and fertility-preserving minimally invasive approach, enabling direct visualization of the uterine cavity and targeted removal of gestational tissue while minimizing myometrial disruption. From a practical clinical standpoint, residual myometrial thickness (RMT) emerges as the key determinant of procedural safety. An RMT ≥ 2 mm appears to allow safe hysteroscopic management under ultrasound guidance alone, whereas an RMT < 2 mm identifies a higher-risk subgroup in whom laparoscopic assistance may serve as a safety adjunct. Therefore, RMT measurement should systematically guide preoperative planning, risk stratification, and intraoperative decision-making. However, given the limited number of reported cases, the variability among studies, and the lack of comprehensive long-term reproductive outcome data, larger prospective studies are needed to further validate this management strategy, better define its safety and efficacy, and confirm its fertility-preserving benefits in a broader patient population.

## Figures and Tables

**Table 1:** Patients’ data.

Author, Year	Patients (*n*)	Age, Year	Obstetric History	Gestation Age, Days	Preoperative *β*-hCG mIU/mL	RMT ^6^	US Findings	Treatment	Rescue Treatment Needed	Success, *n* (%)	Time for β-hCG Normalization (Days)
Katz, 2003 [8]	2	30	NS	56	3467	NS	NS	LPS ^1^ guidance + 8 Fr Karman + HSC ^2^	No	2 (100%)	NS
		28		63	9800				No		
Cai, 2012 [9]	7	32	G4P0	67	3000	NS	57	LPS guidance + 8 Fr Karman + HSC + oxytocin	Yes	4 (57%)	14
		23	G1P0	55	32,000		45		No		35
		31	G3P0	62	3095		65		No		14
		26	G2P1	77	8506		52		No		21
		33	G3P1	61	1400		67		No		28
		NS	NS	NS	NS		NS		Yes		21
		NS	NS	NS	NS		NS		Yes		28
							GS ^3^ (mm)				
Lin, 2013 [10]	1	29	G0 P0	NS	NS	NS	NS	LPS guidance + 18 Fr Karman + HSC	No	1 (100%)	2
Nezhat, 2014 [11]	1	34	G3 P1	63	NS	NS	23 mm CRL HB ^5^ present	LPS milking + Vasopressin + HSC	No	1 (100%)	35
Niu, 2021 [12]	7	28	G1	62	12,967.0	1	30	LPS guidance + 15 Fr Karman + HSC	No	5 (71%)	14
		42	G9P2	51	2682.0	1.2	47		No		63
		35	G5P1	43	3895	1.2	41		No		56
		37	G5P1	50	18,023.8	1.4	36		No		42
		33	G5P0	85	7510.7	1.1	28		No		56
		40	G4P1	42	9381.0	1.2	32		Yes		21
		31	G4P1	42	3505.0	1	18		Yes		42
Nezhat, 2022 [13]	1	33	G2 P0	35	2726	NS ^7^	NS	LPS milking + HSC + suction and curettage	No	1 (100%)	28
Liu, 2025 [14]	1	29	NS	42	3068	4	5 mm GS	US ^4^ guided HSC	No	1 (100%)	NS
Cronin, 2026 [15]	1	20	G2 P1	42	16,840	4	HB present	HSC + US guide suction and curettage	No	1 (100%)	49

^1^ Laparoscopic; ^2^ hysteroscopy; ^3^ gestational sac; ^4^ ultrasound; ^5^ heartbeat; ^6^ residual myometrial thickness; ^7^ not specified.

**Table 2:** Patient Characteristics in the Treatment Failure Group (*n* = 5).

Age (Years, Mean)	Mean Gestational Age (Days)	Mean β-hCG (UI/L, Mean)	Mean RMT (mm, Mean)	Mean GS (mm, Mean)
34, 3	50, 3	5295	1, 1	35, 6

**Table 3:** Patient Characteristics in the Treatment Success Group (*n* = 16).

Mean Age (Years, Mean)	Gestational Age (Days, Mean)	Mean β-hCG (UI/L, Mean)	Mean RMT (mm, Mean)	Mean GS (mm, Mean)
30, 3	56, 5	8479	1, 9	41, 6

## Data Availability

The authors confirm that the data supporting the findings of this study are available within the article.

## Supplementary Materials

The following supporting information can be downloaded at https://www.mdpi.com/article/10.3390/jcm15062158/s1, Table S1: PRISMA Checklist; Table S2: Joanna Briggs Institute (JBI) Critical Appraisal Checklist.

## Abbreviations

The following abbreviations are used in this manuscript:

RMT	Residual Myometrial Thickness
US	Ultrasound
LPS	Laparoscopy
HB	Heartbeat
TACE	Transcatheter Arterial Chemoembolization
MTX	Methotrexate
