# Supplementary material for: The Role of Hysteroscopy for the Treatment of Interstitial Ectopic Pregnancy: A Systematic Review
Source: J Clin Med. 2026 Mar 12;15(6):2158. doi: 10.3390/jcm15062158 (PMC13026084; doi:10.3390/jcm15062158)
Supplement: Supplementary file 1 [file jcm-15-02158-s001.zip › jcm-4166870-supplementary/JBI - Supplementary Table S2.pdf]

Author, Year	Q1	Q2	Q3	Q4	Q5	Q6	Q7	Q8
Katz, 2003	Unclear	Yes	Yes	Unclear	yes	Yes	Yes	Yes
Cai, 2012	Yes	Yes	Yes	Yes	Yes	Yes	Yes	Yes
Lin, 2013	Unclear	Yes	Yes	Unclear	yes	Yes	Yes	Yes
Niu, 2021	Yes	Yes	Yes	Yes	yes	Yes	Yes	Yes
Liu, 2025	Yes	Yes	Yes	Yes	yes	Yes	Yes	Yes
Cronin, 2026	Yes	Yes	Yes	Yes	yes	Yes	Yes	Yes
